# Midline Diastema Closure Following Frenectomy Using M-spring: A Case Report

**DOI:** 10.7759/cureus.65484

**Published:** 2024-07-27

**Authors:** Shefali Singh, Rizwan Gilani, Anjali Kathade, Shrutika Tamgadge, Jay Patil, Aishwarya R Atey, Pratik Rathod

**Affiliations:** 1 Department of Orthodontics and Dentofacial Orthopedics, Datta Meghe Institute of Higher Education and Research, Sharad Pawar Dental College and Hospital, Wardha, IND; 2 Department of Orthodontics and Dentofacial Orthopedics, Annasaheb Chudaman Patil Memorial Dental College, Maharashtra University of Health Sciences, Dhule, IND; 3 Department of Conservative Dentistry and Endodontics, Datta Meghe Institute of Higher Education and Research, Sharad Pawar Dental College and Hospital, Wardha, IND

**Keywords:** m-spring, midline space closure, frenectomy, orthodontics, midline diastema

## Abstract

Midline diastemas are one of the most prevalent dental malocclusions. In young adults this may create aesthetics problems, especially in individuals marked by a gap between central incisors higher than 4 mm. This case report demonstrates the treatment of a patient with Angle's Class I malocclusion and midline diastema with the M-spring appliance resulting in complete closure within four months. The case included a frenectomy for correction of the abnormal labial frenal attachment followed by an M-spring orthodontic appliance. This “M" shaped device, with specific activation points, provides control over tooth movement and consequently achieves this site closure in a short duration of time by tipping the crowns of central incisors in a mesial direction. This procedure aimed to resolve aesthetic issues while also enhancing and ensuring functional occlusion.

## Introduction

A midline diastema is often a normal developmental occurrence. This gap can result from the position of the teeth in their bony crypts, the eruption path of the cuspids, and the enlargement of the premaxilla during the eruption of the maxillary permanent central incisors. Factors such as tooth eruption, migration, physiological readjustment, labial and facial musculature, the anterior force of occlusion, and jaw growth with increased facial muscle tone all contribute to closing the midline gap. The frenum is only considered a problem if it separates the teeth, but natural forces usually close the gap and eliminate the frenum issue. Orthodontic literature identified the superior labial frenum as one of the main causes of midline diastema [1].

Maxillary midline diastema (MMD) is a common dental problem impacting both function and appearance. Broadbent described the normal closure of this gap during the eruption of the maxillary lateral incisors and canines in most children. However, in some individuals, the diastema does not close naturally. In adults, this gap is often viewed as an aesthetic or malocclusion problem. Research indicates that the prevalence of MMD varies significantly with racial background, with higher rates observed in black populations compared to white, Asian, or Hispanic populations [2]. 

Several factors have been suggested as causes for MMD, including discrepancies in tooth or jaw size, abnormal labial frenum attachments, parafunctional habits, tooth loss, periodontal disease, deep bites, and maxillary midline pathologies such as supernumerary teeth. There are also reports of diastema resulting from tongue piercings [3]. The labial frenum can cause a diastema when it inserts into the notch of the alveolar bone, creating a band of dense fibrous tissue between the central incisors. In some cases, the bone around each tooth does not extend to the median suture, causing the central incisors to emerge widely spaced apart, with no bone beneath the frenum. This can result in a V-shaped bone fissure and an abnormal frenum attachment, preventing the midline gap from closing completely as transseptal fibers do not grow across it [3].

To treat such a widely observed dental condition, a specialized orthodontic appliance was designed, i.e., an M-spring. This particular orthodontic gadget is shaped like an M, with three circular loops placed in a unique way: one in the middle and two on either side. M-springs are made of a resilient type of orthodontic wire that when designed to easily toggle and turn on will apply gentle, continuous force closing the diastema over time [2].

Among the main pleasant features of the M-spring is that, it can treat effectively in a shorter duration of time. The use of targeted forces allows the teeth to move gently together, closing the gap. Additionally, this technique is preferred for its simplified nature (using less inventory) and reduced treatment time in comparison to traditional methods [4]. When it comes to managing MMD, orthodontists depend on the precision and versatility of M-spring. Because of its ingenuous design and ease of closing spaces especially cosmetic issues, it is often used for functional and/or aesthetic reasons [5]. This case report depicts the use of M-spring along with frenectomy for early and efficient closure of midline diastema within four months in a young patient.

## Case presentation

A 15-year-old patient reported to the Department of Orthodontics and Dentofacial Orthopedics with a chief complaint of space between the upper front two teeth.

**Figure 1:**
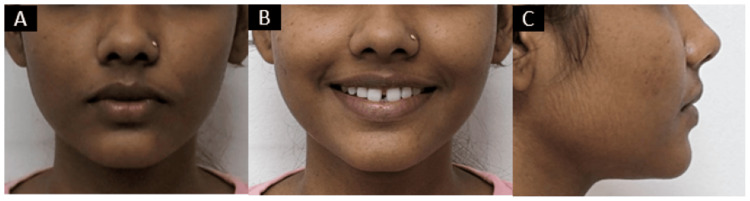
Pretreatment extraoral photographs: (A) frontal, (B) smiling, and (C) profile

Extraoral examination revealed that the patient had an apparently symmetrical face with a mesoprosopic face form and mesocephalic head form. The patient had competent lips and a straight profile. On smiling there was visible space between the upper central incisors (Figure 1).

**Figure 2:**
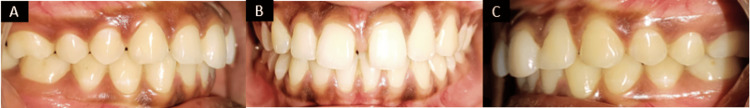
Pretreatment intraoral photographs: (A) right occlusion, (B) frontal, and (C) left occlusion

Intraoral examination revealed a full permanent dentition. An adequate zone of attached gingiva was present, with satisfactory gingival health. All permanent teeth had erupted except the third molars in all four quadrants. Upright incisors were seen in the upper arch anterior spacing of 4 mm between 11 and 21 and a high frenal attachment. Both maxillary and mandibular arches were U-shaped with Angle’s Class I molar and canine relationship on both sides. Overjet and overbite were 4 mm and 3 mm, respectively (Figure 2).

Functional examination showed normal speech pattern, oro-nasal breathing, and matured swallowing pattern. The mandible closure path was deviated toward the left side, and there were no associated signs and symptoms of temporomandibular disease.

Orthodontic phase

**Figure 3:**
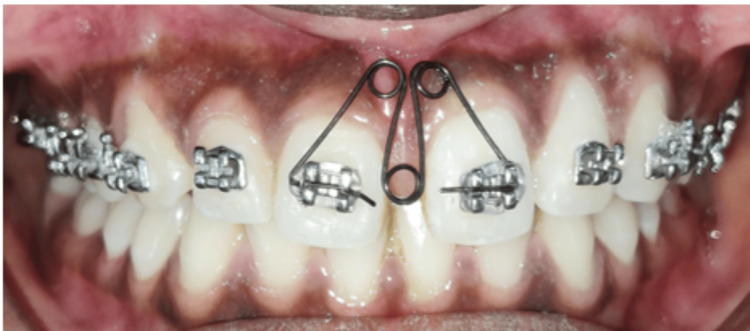
Intraoral photograph depicting placement of M-spring

Before beginning with orthodontic treatment, a frenectomy was performed to remove the highly positioned upper frenum. Bonding of the upper arch using the MBT 0.022” slot was done. M-spring was chosen as a treatment modality and was given immediately after bonding of the upper arch. Fabrication of the M-spring was done by 0.016” AJ Wilcock wire. The M-spring was made up of three loops, each around 3-4 mm in diameter. The middle loop was situated in the middle of the three loops, while the other two were situated next to it. The location of the labial loops was 5-6 mm above the maxillary bracket. The active arm is bent inward by 45 degrees during activation to ensure that it fits entirely inside the bracket slot. This is required in order to insert the active arm into the slot (Figure 3).

**Figure 4:**
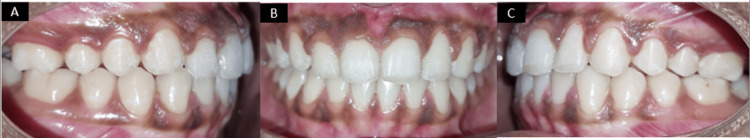
Post-treatment intraoral photographs: (A) left occlusion, (B) frontal, and (C) right occlusion

The midline diastema was closed in three months, but there was space created between the lateral and central incisors due to the tipping of the central incisors. Continuous archwires were given in the upper arch and minor space corrections were made using an elastomeric chain on 0.017×0.025 stainless steel wire. After space closure Class I molar and canine relation was maintained (Figure 4).

**Figure 5:**
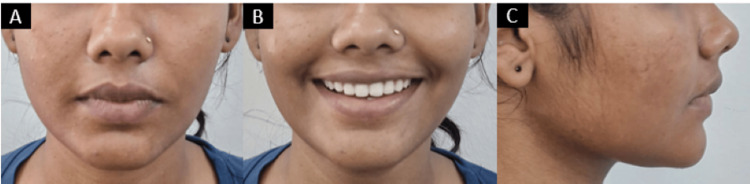
Post-treatment extraoral photographs: (A) frontal, (B) smiling, and (C) profile

This treatment modality achieved good results within a smaller amount of time while maintaining a straight profile and taking care of the esthetic concern of the patient by improving her smile (Figure 5).

## Discussion

Midline diastema is one of the most common types of malocclusions that young people find very unattractive. The appearance of this space occurs due to different dental sizes, abnormal tongue positioning, discrepancies in tooth size and jaw dimensions, congenitally missing lateral incisors, peg-shaped laterals, midline pathologies, and habits among others [6].

Orthodontic treatment often involves the use of different metals to effectively manage midline diastema and achieve a natural, aesthetically pleasing smile. The choice of metals varies based on their properties and cross-sectional characteristics, which are crucial for controlling treatment outcomes and preventing relapse. This article specifically discusses a case treated with an "M" spring, highlighting its role in addressing midline diastema [7].

M-springs are designed to provide controlled forces for the movement of teeth, making them suitable for diastema closure. Previous studies have shown that M-springs can generate appropriate orthodontic forces that are effective in bringing the central incisors closer together without causing significant discomfort to the patient. The shape of the M-spring allows for both labial and lingual forces, which ensures that the teeth move in a desired direction, reducing the midline gap effectively [6].

Compared to other methods, such as clear aligners, braces, and elastic bands, M-springs offer a more focused and cost-effective solution for diastema closure. Braces, while effective, often require a longer treatment period and can be uncomfortable. Clear aligners, though aesthetically pleasing, may not provide targeted force as effectively as M-springs. Elastic bands are often less predictable in their outcomes and may require more frequent adjustments [8].

Patient compliance is a critical factor in the success of orthodontic treatments. Studies have indicated that M-springs, due to their simplicity and ease of use, have a higher compliance rate among patients [8,9]. The minimal discomfort associated with M-springs compared to other orthodontic appliances also contributes to their preference among patients, particularly in adult orthodontics where esthetic concerns are paramount [9].

While M-springs are effective, it is crucial to consider the patient’s dental anatomy and the cause of the diastema. For instance, a midline diastema caused by a thick or low-attached labial frenum may require frenectomy in conjunction with orthodontic treatment for optimal results [10].

## Conclusions

M-springs offer a reliable, efficient, and patient-friendly method for closing midline diastema. Their targeted application, combined with high patient compliance and comfort, makes them a preferred choice in orthodontic treatment. Comprehensive diagnosis and personalized treatment planning are crucial to achieving the best outcomes. Future research should focus on long-term stability and the comparative effectiveness of M-springs with emerging orthodontic technologies to further validate their use in clinical practice. This discussion highlights the practical advantages and considerations of using M-springs for midline diastema closure, providing a comprehensive understanding for both clinicians and patients.
